# Web-Based Apps for Responding to Acute Infectious Disease Outbreaks in the Community: Systematic Review

**DOI:** 10.2196/24330

**Published:** 2021-04-21

**Authors:** Emma Quinn, Kai Hsun Hsiao, Isis Maitland-Scott, Maria Gomez, Melissa T Baysari, Zeina Najjar, Leena Gupta

**Affiliations:** 1 Sydney Local Health District Camperdown Public Health Unit Royal Prince Alfred Hospital Campus Camperdown, Sydney, NSW Australia; 2 School of Public Health Faculty of Medicine and Health University of Sydney Camperdown, Sydney, NSW Australia; 3 Charles Perkins Centre Faculty of Medicine and Health University of Sydney Camperdown, Sydney, NSW Australia

**Keywords:** software, mHealth, infectious diseases, outbreaks, mobile apps, mobile phone, eHealth

## Abstract

**Background:**

Web-based technology has dramatically improved our ability to detect communicable disease outbreaks, with the potential to reduce morbidity and mortality because of swift public health action. Apps accessible through the internet and on mobile devices create an opportunity to enhance our traditional indicator-based surveillance systems, which have high specificity but issues with timeliness.

**Objective:**

The aim of this study is to describe the literature on web-based apps for *indicator-based surveillance and response to acute communicable disease outbreaks* in the community with regard to their design, implementation, and evaluation.

**Methods:**

We conducted a systematic search of the published literature across four databases (MEDLINE via OVID, Web of Science Core Collection, ProQuest Science, and Google Scholar) for peer-reviewed journal papers from January 1998 to October 2019 using a keyword search. Papers with the full text available were extracted for review, and exclusion criteria were applied to identify eligible papers.

**Results:**

Of the 6649 retrieved papers, 23 remained, describing 15 web-based apps. Apps were primarily designed to improve the early detection of disease outbreaks, targeted government settings, and comprised either complex algorithmic or statistical outbreak detection mechanisms or both. We identified a need for these apps to have more features to support secure information exchange and outbreak response actions, with a focus on outbreak verification processes and staff and resources to support app operations. Evaluation studies (6 out of 15 apps) were mostly cross-sectional, with some evidence of reduction in time to notification of outbreak; however, studies lacked user-based needs assessments and evaluation of implementation.

**Conclusions:**

Public health officials designing new or improving existing disease outbreak web-based apps should ensure that outbreak detection is automatic and signals are verified by users, the app is easy to use, and staff and resources are available to support the operations of the app and conduct rigorous and holistic evaluations.

## Introduction

### Background

Despite global progress in improving environmental health, household living conditions, vaccination coverage, and medical treatments, communicable diseases remain a significant threat to public health and emergency preparedness and are among the biggest contributors to disease and disability worldwide [1]. Factors such as climate change, population growth, global travel and trade, and persistent social inequalities further contribute to the potential risks and impacts of emergent or re-emergent communicable disease outbreaks [2-5].

It is well recognized that the earlier outbreak containment and response actions are initiated, the greater the potential for these measures to reduce attack rates, disease spread, and overall morbidity and mortality. The outbreak of severe acute respiratory syndrome in China in early 2003 provides a good example of the effectiveness of detection and outbreak containment measures, that is, isolation and quarantine, in reducing the spread of a disease [6,7]. A more contemporary example is the early implementation of enhanced surveillance and proactive case finding for COVID-19 in Taiwan, where, to date, case numbers remain comparatively low despite Taiwan’s proximity to China, high inter-Strait trade and travel, and early entry into the pandemic [8,9]. A rapid and effective response to a communicable disease outbreak is a complex process reliant on early recognition of an aberrant disease pattern (compared with some baseline or *normal* activity), with notification and verification of the cluster or outbreak important before containment is initiated.

Historically, country-level indicator-based surveillance systems have involved paper-based or phone notifications of communicable diseases under respective local public health legislation and International Health Regulations [10-13]. Although electronic laboratory reporting has improved the timeliness of this type of surveillance system, inherent delays in case notification result in time delays in the detection of aberrant patterns and subsequent outbreak containment [14,15]. From the early 1980s, there has been investment in *early warning systems* such as syndromic surveillance systems that collect, analyze, and detect unusual signals related to a syndrome (ie, a group of symptoms) or *event-based systems* that capture and analyze internet-based or *rumor surveillance* data to detect public health risks [16-21]. These systems have complemented, rather than replaced, traditional indicator-based systems. Although event-based or early warning systems can detect unusual patterns of communicable diseases earlier than traditional indicator-based systems, the mathematical algorithms used to support accurate and valid signal detection are still controversial, and the filtering of statistical signals into truly meaningful public health risk alerts requires significant input or moderation [16,17].

The field of communicable disease surveillance has evolved markedly over the past few decades in terms of digital systems, software, and accessibility, particularly with the rapid evolution of the internet [22]. Apps accessible through the internet and on mobile devices are increasingly being used to monitor the health and well-being of individual clients or users and by public health staff to track and monitor population epidemiology [23-25]. These technological developments present an opportunity to modernize traditional paper- and indicator-based surveillance systems using functions such as digitized data entry and storage; automated outbreak detection; and real-time case reporting, analysis, and alert notifications [17]. In addition, the wider accessibility (in terms of both physical access and ease of use) of web-based, or mobile-based apps in particular, can improve the awareness and ability of users to participate in surveillance activities [24].

Although improving the timeliness and sensitivity of surveillance activity is a worthwhile goal in outbreak management, there is a growing opportunity to use web-based apps to help deliver response actions. Examples include automated alerts to key responders about actions needed, links to guidelines and resources, and checklists to guide field staff action on the ground. The Integrated Disease Surveillance and Response strategy from the World Health Organization (WHO) recognizes the importance of scaling up electronic surveillance systems to respond to infectious disease outbreaks [26]. An example of the real-time use of a web-based surveillance system to facilitate an outbreak response during a public health emergency is the Chinese system Decision Support System for Response to Infectious Disease Emergencies tested during the H1N1 pandemic [27]. Although examples of these systems appear increasingly in the published literature, the reporting on their design, implementation, and evaluation of these systems is highly variable. In contrast to previous systematic reviews [16,17,28,29], we aimed to systematically review the literature describing web-based apps for *indicator-based surveillance and response to acute communicable disease outbreaks* in the community with regard to their design, implementation, and evaluation.

### Objectives

The three key objectives of this review were to:

1. Identify and describe the mobile and web-based apps that use surveillance data to respond to acute communicable disease outbreaks in the community.
2. Identify key lessons learned for the design and implementation of these apps.
3. Identify any methods used to evaluate the effectiveness of these apps.

We hope that our review will inform the effective development and use of these apps from a health system perspective [30].

## Methods

### Scope of This Review

The scope of this review is defined here, as the evidence in this area is rapidly emerging and technically focused; therefore, we felt the need to clarify the terms and definitions used throughout this review. This review focuses on software apps that collate and analyze communicable disease outbreak data. We defined *software apps* as sequential operating programs that instruct the functioning of a digital computer. These software apps may be web based or mobile based and are accessible via devices such as mobile phones and other smart devices (also known as mobile health [mHealth] or mobile apps) and desktop or laptop computers [23].

We defined an *outbreak* as the occurrence of cases of disease in excess of *normal pattern*, with cases linked in place and time as demonstrated by epidemiological or laboratory data. The number of cases defining an outbreak varies according to the disease-causing agent and context. We targeted acute (epidemic not endemic) outbreaks in a community setting (ie, not nosocomial outbreaks). Importantly, we specifically considered confirmed outbreaks using indicator-based surveillance data. Indicator-based surveillance data require defined counts of cases and contacts (as per national case definitions) using clinical, laboratory, and epidemiological information to define and monitor an outbreak. Thus, papers describing apps used for early warning, syndromic, or event-based (rumor or internet) surveillance were outside the scope of this study. We used *response* in this study to specifically refer to the detection and notification of the outbreak to the appropriate public health authority and initiation of interventions to help control the spread and impact of the outbreak, for example, outbreak investigation, cohorting, isolation and quarantine, infection control, treatment, and prophylaxis and vaccination. Papers were excluded if the app collected data without the explicit capacity to trigger a specific outbreak notification to a public health authority for further investigation. Finally, we considered app effectiveness in this context to comprise 2 things: (1) end users’ measured or self-reported ease, comfort, and ability to use the technology for its intended purpose and (2) measured ability of the app to meet its intended goals/objectives, for example, increased sensitivity, specificity, or timeliness in outbreak detection.

### Search Methodology

A systematic review of the literature was conducted using the PRISMA (Preferred Reporting Items for Systematic Reviews and Meta-Analyses) framework [31] (Multimedia Appendix 1) to identify eligible papers for review. A search of 3 web-based databases was conducted: (1) MEDLINE via OVID, (2) Web of Science Core Collection, and (3) ProQuest Science for peer-reviewed journal papers (ie, case studies, case reports, original papers, and reviews) from January 1998 to October 2019 using the following search terms as *keywords* where databases allowed or selected as *anywhere in the paper*:

- (((smartphone OR android OR software OR system OR computer OR website OR web OR application OR app) AND
- (infectious disease OR communicable diseases) AND
- (outbreak)))

Limits to the search were also applied, including only papers published in English, human subjects (if appropriate), and full text available. Google Scholar was also searched using a multifield keyword search using the terms listed earlier for the years between 1998 and 2019 [32]. As Google provides search results listed by relevance, only papers listed in the first 10 pages of the Google search were retrieved for review.

Papers obtained via our database and Google search were excluded if they:

1. Did not describe a web-based, mobile-based, or phone-based app or software associated with managing an acute human infectious disease outbreak in the community
2. Described apps or software used solely for management during an outbreak after it had already begun (ie, without function of disease surveillance and/or outbreak detection), including monitoring drug or vaccine therapy and/or effectiveness and mapping of cases
3. Described apps or software used for modeling, estimating, or simulating infectious disease outbreak responses only (eg, not used to monitor real-time events) or using geographic information systems to model spatial distributions and patterns
4. Described apps or software for surveillance and detection (both retrospective and prospective) of infectious disease outbreaks but did not report directly to a public health organization or workforce or trigger any explicitly stated public health outbreak control action in response
5. Described apps or software exclusively focused on early warning, syndromic, or event-based surveillance.

Citation searches were also performed by checking the reference lists of the included papers to identify any new relevant papers not captured by our original searches [33,34].

### Data Extraction and Synthesis

Paper titles and abstracts were independently screened by 3 of the authors (EQ, IS, and KH), using the exclusion criteria to identify a list of papers for full-text review. The same 3 authors independently screened the full-text papers against the exclusion criteria. Two authors (EQ and IS) extracted the following information from each paper: (1) overview of the purpose of the app, (2) setting (location where the app was mainly used, eg, national to local public health offices or in field-based locations), (3) mechanisms for detecting outbreaks (eg, algorithmic and/or statistical models), (4) features to support outbreak response (eg, notification to key responders, advice on outbreak investigation, information on how to conduct contact tracing, implement infection control, or targeted education resources), (5) lessons learned from app development or implementation (as described by each paper’s authors and extracted by EQ and IS for the entire paper), and (6) evaluation methods and effectiveness of apps. Data extracted from all included papers were discussed and agreed upon by the authors (EQ and IS) before reporting. Lessons learned from the development or implementation of the apps were further classified into 3 categories: (1) technical (factors related to app features and functions), (2) personal or social (factors related to the users of the app), and (3) organizational (factors related to the owning organization of the app). These categories are consistent with those used by Cresswell et al [35] and Gagnon et al [36] to classify themes in relation to health care technology adoption.

## Results

### Search Results

**Figure 1:**
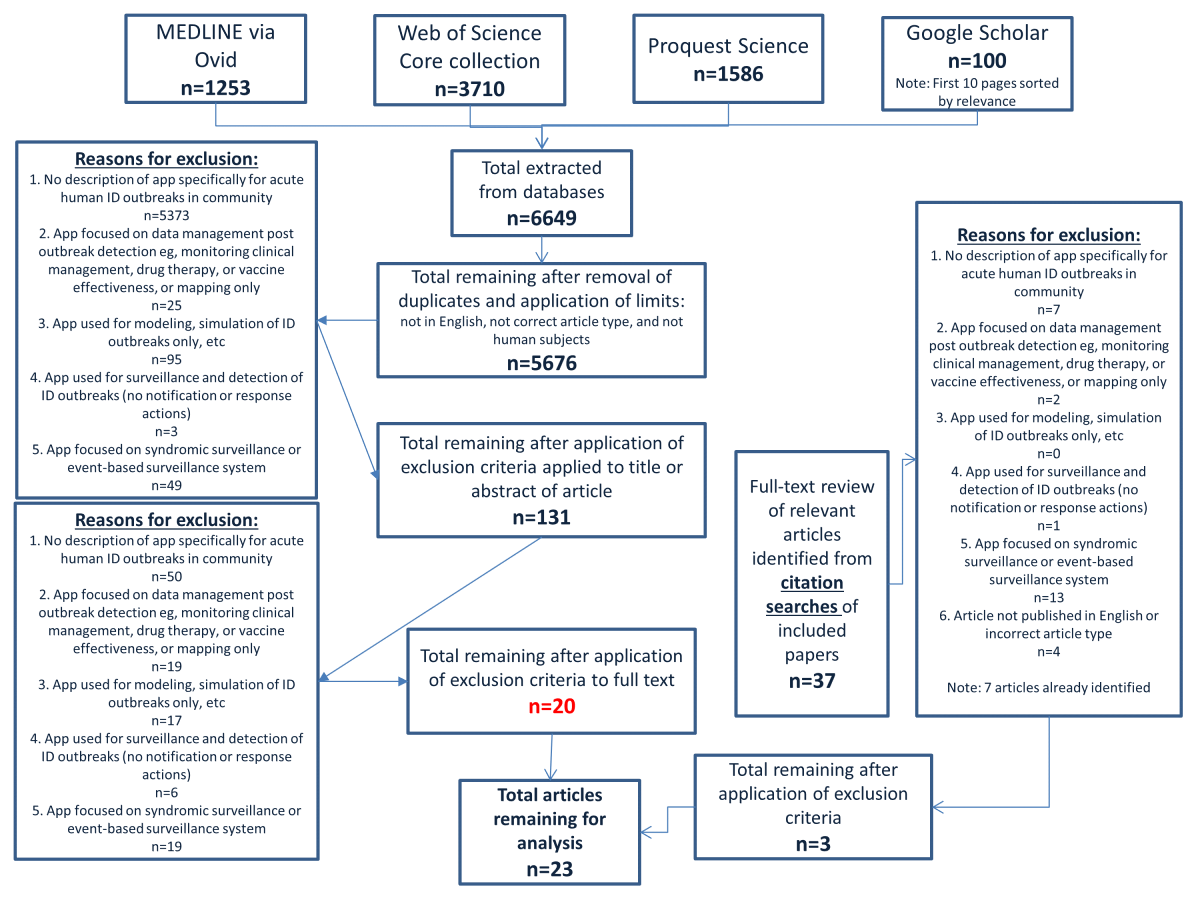
Systematic search strategy results. ID: infectious diseases.

The search (Figure 1) generated 6649 papers, with 5676 papers remaining after removal of duplicates and application of limits, as described earlier. Of these, 5545 were excluded based on title and abstract and 131 remained for full-text review (Figure 1). After full-text review, 111 papers were excluded and 20 were included (Figure 1). An additional 3 papers were identified via our citation search (Figure 1). In total, 23 papers describing 15 apps were included in this study (Table 1). The majority (20/23, 87%) were descriptive in nature, including 1 review paper [37] describing several apps. Only 3 papers were empirical studies that provided comparative outcomes before and after implementation [38-40], with 1 of these studies also using an adjacent district as a control [38] (Table 1). Of the 23 papers, 19 described web-based apps that were implemented [27,37,39-55], 3 described apps that were being piloted [38,56,57], and 1 described a web-based app in development [58]. The unit of analysis for reporting the results in this review is the number of web-based apps (n=15), as some papers described multiple apps.

**Table 1:** Summary of included papers.

App name and reference			Study type		Stage of app development or implementation (as described in paper)
**Computer-Assisted Outbreak Detection** **—** **SmiNET**					
	Cakici et al (2010) [41]	Descriptive		Implemented (currently in routine use at Swedish Institute for Infectious Disease Control)	
	Kling et al (2012) [42]	Descriptive		Implemented	
	Rolfhamre et al (2006) [43]	Descriptive		Implemented	
**Argus**					
	El-Khatib et al (2018) [38]	Empirical (before and after+adjacent district control)		Piloted (15 weeks)	
**SurvNet @Robert Koch Institute**					
	Faensen et al (2006) [44]	Descriptive		Implemented (used at local, state, and national levels)	
	Hulth et al (2010) [37]	Review - descriptive		Implemented	
	Krause et al (2007) [45]	Descriptive		Implemented	
	Salmon et al (2016) [46]	Descriptive		Implemented	
	Straetemans et al (2008) [47]	Descriptive		Implemented	
**Integrated Crisis Alert and Response System**					
	Groeneveld et al (2017) [56]	Descriptive		Piloted (using 3 syndromes)	
**Vesuv**					
	Guzman-Herrador et al (2016) [48]	Descriptive		Implemented	
**Statens Serum Institut automated outbreak detection system**					
	Hulth et al (2010) [37]	Review - descriptive		Implemented	
**National Institute for Public Health and Environment (RIVM^a^** **) automated surveillance**					
	Hulth et al (2010) [37]	Review - descriptive		Implemented	
**Early Warning and Response System**					
	Karo et al (2018) [49]	Descriptive		Implemented	
	Sheel et al (2019) [50]	Descriptive		Implemented	
**Decision Support System for Response to Infectious Disease Emergencies**					
	Li et al (2013) [27]	Descriptive		Implemented	
**China Infectious Diseases Automated Alert and Response System**					
	Li et al (2014) [39]	Empirical (before and after)		Implemented	
	Yang et al (2011) [51]	Descriptive		Implemented	
	Zhang et al (2014) [52]	Descriptive		Implemented	
**Public Health Emergency Response Information System**					
	Liang et al (2004) [58]	Descriptive		In development	
**WHONET** **—** **SaTScan**					
	Stelling et al (2010) [53]	Descriptive		Implemented	
	Vinas et al (2013) [57]	Descriptive		Piloted (participating laboratories in select provinces)	
**French Institute for Public Health Surveillance app**					
	Vaux et al (2009) [54]	Descriptive		Implemented	
**Infectious Disease Surveillance System**					
	Widdowson et al (2003) [55]	Descriptive		Implemented	
**Adjustable Epidemiologic Information System**					
	Wu et al (2011) [40]	Empirical (before and after)		Implemented	

^a^RIVM: Rijksinstituut voor Volksgezondheid en Milieu; English name or translation is National Institute for Public Health and Environment.

### Overview of App Purpose

As shown in Multimedia Appendix 2 [27,37-64], the most commonly stated purpose of the 15 web-based apps described in the 23 included papers was to improve the early detection of infectious disease outbreaks (8/15 apps) [38,39,41-43,48,51,52,54-57], predominantly by improving the timeliness of reporting, thereby enabling a rapid response. Other app purposes include automatic outbreak detection, usually involving complex statistical modeling on routinely collected notifiable disease data to determine if thresholds for an outbreak were met (4/15 apps) [37,53,55] and enhanced surveillance for infectious disease outbreaks during emergencies (3/15 apps) [49,50,58].

### Setting and Location of Web-Based App Use

As shown in Multimedia Appendix 2, most of the 15 web-based apps were targeted at multiple users from across public health authorities or government departments (12/15 apps) [27,37-48,51-53,55,57,58]. A total of 8 apps were designed for users from national, regional, and local public health authorities [27,38,40-43,48,53,55,57,58], and 4 apps focused on surveillance and reporting at the national level only [37,39,44-47,51,52]. A total of 3 apps were designed for use at the community level [49,50,54,56], either in general practice clinics and hospitals, sentinel facilities, field-based locations, or nursing homes. Of the 15 web-based apps, 8 were used in the European Union [37,41-48,54-56]; 3 in China [27,39,51,52,58]; and the remainder in the Central African Republic [38], Fiji and Myanmar [49,50], Argentina [53,57], and Taiwan [40].

### Mechanisms for Detecting and Responding to Outbreaks

Outbreak detection functionality [37,39-48,51-57] was specifically described for 11 web-based apps, with all of these using some form of algorithmic detection of outbreaks, usually based on historical data (Multimedia Appendix 2).

A total of 8 other apps [37,39,41-47,51-53,55-57] also had in-built statistical capability to model and detect outbreaks based on whether the disease activity had exceeded *normal levels* (Multimedia Appendix 2). The most common model used was by Farrington et al [59], followed by SaTScan [60] and Stroup et al [61]. For all 15 web-based apps, the outbreak response functionality was limited to email or SMS notifications of outbreak detection to public health authorities for further follow-up and investigation (Multimedia Appendix 2).

### Lessons Learned From the Development and Implementation of the Apps

#### Technical

The 2 most common lessons learned (Table 2) relating to a technical aspect of apps, as reported by the authors of the papers [37,39,41-48,51,52,55,56], were the need to ensure outbreak detection was automatic (ie, real-time and proactive without human involvement) and that signals were verified by users (ie, to ensure action was initiated). This was central to the *early outbreak detection* function of the apps [37,56]. Associated with this, however, is the issue of false-positive outbreak signals, which were mentioned across 6 web-based apps [37,39,41-47,49,51,52,55,56]. Outbreak detection methods that yield a low positive predictive value increase the number of outbreak signals, which, in turn, increases the workload for public health staff in reviewing and responding to these signals. Authors suggested that having standard operating procedures to detail how users or staff should respond to outbreak signals would not only potentially reduce workload but also ensure no signal is missed [37,44-47] (Table 2). *Flexibility and ease of use of the app* were also frequently mentioned (10 times across 5 apps) [37,38,41-47,49,50,53,55,57], and this specifically included using open-source or off-the-shelf software to promote web-based collaborative development of the app, simple data entry forms that could be tailored to disease groups, and flexible detection algorithms that were configurable to the epidemiology of the disease, for example, low- versus high-incidence condition (Table 2). Ensuring the *confidentiality and security of information within the app* [27,56] and *integration with other existing software* [37,41-43] (Table 2) were also mentioned as important in maintaining appropriate use of the app.

**Table 2:** Summary of technical, personal, and organizational lessons learned from development and implementation of web-based apps for infectious disease outbreak response.

Lessons learned			Number of mentions and number of apps		References
**Technical**					
	Ensure detection methods are automatic and signals are verified by users	12 mentions; 8 apps		Hulth et al (2010) [37]; Li et al (2014) [39]; Cakici et al (2010) [41]; Kling et al (2012) [42]; Rolfhamre et al (2006) [43]; Faensen et al (2006) [44]; Krause et al (2007) [45]; Salmon et al (2016) [46]; Straetemans et al (2008) [47]; Guzman-Herrador et al (2016) [48]; Yang et al (2011) [51]; Zhang et al (2014) [52]; Widdowson et al (2003) [55]; Groeneveld et al (2017) [56]	
	Ensure flexibility and ease of use	10 mentions; 5 apps		Hulth et al (2010) [37]; El-Khatib et al (2018) [38]; Cakici et al (2010) [41]; Kling et al (2012) [42]; Rolfhamre et al (2006) [43]; Faensen et al (2006) [44]; Krause et al (2007) [45]; Salmon et al (2016) [46]; Straetemans et al (2008) [47]; Karo et al (2018) [49]; Sheel et al (2019) [50]; Stelling et al (2010) [53]; Widdowson et al (2003) [55]; Vinas et al (2013) [57]	
	Maintain security	2 mentions; 2 apps		Li et al (2013) [27]; Groeneveld et al (2017) [56]	
	Ensure the app integrates with other software	2 mentions; 2 apps		Hulth et al (2010) [37]; Cakici et al (2010) [41]; Kling et al (2012) [42]; Rolfhamre et al (2006) [43]	
**Personal**					
	Increase user awareness and engagement with the app	2 mentions; 2 apps		Hulth et al (2010) [37]; Faensen et al (2006) [44]; Krause et al (2007) [45]; Salmon et al (2016) [46]; Straetemans et al (2008) [47]; Guzman-Herrador et al (2016) [48]	
**Organizational**					
	Develop and maintain resources for operational support of the app	13 mentions; 6 apps		Li et al (2013) [27]; Hulth et al (2010) [37]; El-Khatib et al (2018) [38]; Li et al (2014) [39]; Faensen et al (2006) [44]; Krause et al (2007) [45]; Salmon et al (2016) [46]; Straetemans et al (2008) [47]; Guzman-Herrador et al (2016) [48]; Karo et al (2018) [49]; Sheel et al (2019) [50]; Yang et al (2011) [51]; Zhang et al (2014) [52]	
	Conduct rigorous evaluations of the app	7 mentions; 5 apps		Hulth et al (2010) [37]; Wu et al (2011) [40]; Cakici et al (2010) [41]; Kling et al (2012) [42]; Rolfhamre et al (2006) [43]; Faensen et al (2006) [44]; Krause et al (2007) [45]; Salmon et al (2016) [46]; Straetemans et al (2008) [47]; Vaux et al (2009) [54]; Groeneveld et al (2017) [56]	
	Education and training	4 mentions; 2 apps		Hulth et al (2010) [37]; El-Khatib et al (2018) [38]; Faensen et al (2006) [44]; Krause et al (2007) [45]; Salmon et al (2016) [46]; Straetemans et al (2008) [47]	
	Coverage of uptake of the app	3 mentions; 3 apps		Hulth et al (2010) [37]; Widdowson et al (2003) [55]; Groeneveld et al (2017) [56]	

#### Personal

The main lesson learned relating to users of apps was the need to *maintain user awareness or engagement with the app* (2 mentions across 2 apps) [37,44-48] (Table 2). There was a distinct lack of themes related to the environment of the user, for example, culture or governance of the organization that the user works in, which can impact technology adoption [36].

#### Organizational

At the organizational level, the most frequently reported lesson learned was the need to *develop and maintain operational resources to support the use of the app* (13 mentions across 6 apps) [27,37-39,44-52] (Table 2). This included having not only staff with the necessary skills to ensure adequate governance of the app from an information technology (IT) perspective but also staff to train users and assist with implementation of the app within field or sentinel sites, for example, user profile management [27,38,48]. In addition, organizations that implement the app need to ensure there is adequate IT infrastructure and potentially Wi-Fi or mobile reception to support use [49]. There were also 7 mentions across 5 apps [37,40-47,54,56] (Table 2) of the need to *conduct comprehensive and rigorous evaluations of the use and effectiveness of these apps*, both from a user-based design perspective and to ensure the app was meeting its goals and objectives, that is, detect and respond to outbreaks.

### Evaluation Methods and Reported Effectiveness of Web-Based Apps

Papers on 6 out of 15 apps reported evaluation data [37-39,44-47,50-52]. Most evaluations were cross-sectional studies reporting effectiveness of the apps for detecting outbreaks, compared with paper-based or routine methods, that is, sensitivity of detection or time from detection of an outbreak to notification of public health staff.

Other than the evaluation conducted by Sheel et al [50], which used the surveillance system evaluation criteria of the Centers for Disease Control and Prevention [63], there were no user-based evaluations, for example, measuring user satisfaction. The evaluation by El-Khatib et al [38] was the only empirical study comparing 15 weeks of pilot data from the app before (2015) and after (2016) the study in a pilot district, that is, Mambere-Kadei, compared with a control district, that is, Nana-Mambere in the Central African Republic. They found that the median completeness of weekly reports significantly improved in the pilot district over time and in comparison with the control district (81% in 2016 vs 29% in 2015 for Mambere-Kadei and 52% for Nana-Mambere in 2016; *P*<.01). An overall significant reduction in time to reporting was observed in the Mambere-Kadei district over the pilot period (Kaplan-Meier survival analysis; *P*<.01). However, no evaluation study measured the effectiveness of implementation of the apps (for future scale-up and use) or more proximal health outcomes related to decreased response time, for example, attack rates, hospitalization rates, and death rates from outbreaks captured by the apps.

## Discussion

### Principal Findings

This systematic review summarizes the features of indicator-based surveillance web-based apps for acute infectious disease outbreak detection and response and uniquely reports on lessons learned from their development and implementation and evaluation of these apps. Our review identified 23 papers describing 15 web-based apps [27,37-58], the majority of which were developed to improve the early detection of infectious disease outbreaks, targeting government settings and experienced public health staff, and comprising complex algorithmic and/or statistical outbreak detection mechanisms.

Most web-based apps identified in this study were designed for government public health staff (usually at the national or regional level) who, in their capacity to collect surveillance data under relevant public health legislation, use these web-based apps to better coordinate outbreak detection and notification. This is not surprising, as there has been an impetus over recent decades toward harnessing web-based apps and, more recently, cloud computing to better facilitate surveillance of infectious diseases to improve upon the capacity and timeliness of paper-based systems [65].

In addition to improving outbreak detection, our review identified the need for web-based apps to have features that support secure information exchange and analysis [37-49,51,52,54,56,58]. For example, this included dashboard functionality (ie, visual display of outbreak data), capability to distribute bulletins, or integration with other statistical software for analysis. However, as identified in this study and other studies, web-based app features that directly support response activities to outbreaks on the ground (eg, outbreak action checklists and notifications of remaining response actions) are lacking [66]. A systematic review of 58 mHealth apps used in Africa to aid the response to the 2014-2015 Ebola outbreak revealed that very few had functionality to support surveillance, case management and contact tracing, or reporting on infection control measures and few were designed with both medical and public health users in mind [67].

Another common theme is the need for web-based apps to be user-centric in their design to enhance adoption, uptake, and use [36]. The authors have recommended mixed methods research be used in user-based design to elucidate the scenario, tasks, workflows, and user characteristics that can influence the success of an app [66]. There are a growing number of validated evaluation tools and frameworks to help assess user engagement and usability [68] with web-based apps, for example, user version of the Mobile Application Rating Scale tool [69]. However, this study identified no current evidence of the use of these user-based evaluation frameworks in the development of web-based apps for acute infectious disease responses. It must be noted that there was a distinct lack of analysis of the environment of the user (eg, culture or governance of their organization), which can be important to understand in terms of technology adoption [36].

Our review identified evaluation studies showing that some of these web-based apps can reduce the time to detection and notification of infectious disease outbreaks [38-40]. Improvements in the timeliness of outbreak detection are likely because of app features that support automated outbreak detection and notification, that is, statistical models that analyze complex data quickly to determine if a disease activity is above *normal* and then automatically notify the right public health staff at the right time. Authors publishing other reviews on the evaluation of prospective statistical methods for detection of outbreaks highlight the need for more rigorous and comprehensive evaluation of detection methods, for example, using larger dummy data sets and/or simulated outbreaks, clearly defined evaluation indicators (sensitivity, specificity, and timeliness), and multiple statistical techniques (eg, cumulative sum vs space-time permutations vs geospatial regression analysis) [70,71]. Evidence shows that epidemic features of outbreaks affect the performance of detection methods, for example, low incidence conditions or baseline counts and seasonality [72]. The authors of the papers in this study also reported the need for standard operating procedures to ensure signals were verified by staff to reduce the low positive predictive value or false positivity rate and subsequent workload [37,44-47].

Implementation science is a growing field of research dedicated to understanding the factors necessary for the real-world implementation of health interventions [73]. This study identified lessons learned that mostly focused on technical and organizational factors. These included outbreak verification processes, staff, and resources to support operations. These organizational factors are consistent with those identified in a systematic review of the implementation of eHealth interventions (not web-based apps per se), which also revealed that implementation issues appear consistent over the past decade or so (eg, issues with funding, infrastructure, policies and standards, interoperability) [30]. The growing number of web-based apps being used in infectious disease control demands evidence from an implementation science perspective and at all levels (user, system, and organization) to ensure that investment in these new technologies provides a cost benefit for the owning organization in the long term.

This study also highlights a distinct lack of evaluation studies for web-based apps of this kind. Only 6 of 15 apps identified in this study had been evaluated, and evaluations were focused on the technical aspect of improving the timeliness and sensitivity of detection of outbreaks, rather than other forms of effectiveness evaluation, for example, user needs assessment or health outcomes evaluation. Previous authors have recommended clear definitions of the processes that impact the timeliness of reporting (ie, implementation factors) and how timeliness is defined and measured by the owning organization [74,75]. However, other forms of evaluation should also be considered. As mentioned by Calba et al [76], the sociological and economic impacts of technology are important. Researchers should also use validated tools or frameworks where possible and ensure that the evaluation is tailored toward the defined attributes, processes, and context of the surveillance system. Researchers involved in the CONSORT-EHEALTH group have developed a unified checklist for reporting evaluations of web-based and mHealth apps that is currently limited to controlled trials [77]. Until further advice is available, we recommend that researchers take note of the need to think more holistically about the evaluation of web-based apps for infectious disease outbreak responses.

### Limitations

As is the case with other systematic reviews, our review process was limited by the breadth of the published literature. There may be many more eligible outbreak detection and response apps that have been developed or implemented but have not been published. Publication bias in this subject area likely skews toward apps that have been successfully developed or implemented, as is the case with all the papers found and included in this study [78].

This study is also limited by the lack of a widely applied, standardized terminology for describing the types and functions of different digital health technologies; only recently has such a standardized taxonomy been proposed by the WHO in recognition of this challenge [79]. Thus, although we included as many synonyms or related search terms for *app* as conceivable in an attempt to apply a sensitive and comprehensive search methodology, there may still be published papers on relevant apps that have not been identified in this study.

Finally, the lessons extracted from the papers were based on the reported perspectives and experiences of their academic authors. The extent of the involvement and visibility of these authors in full app development or implementation processes is unclear. As such, the reported lessons may be biased toward more proximal insights derived from the late implementation or evaluation stages, missing important lessons relating to app development or initial implementation.

### Conclusions

Digital health technologies, such as web- and mobile-based apps, present unique and beneficial opportunities for timely and effective responses to communicable disease outbreaks. This has certainly been underscored by the rapid digital innovation and implementation in response to the current COVID-19 pandemic, the most visible of which are mobile contact tracing apps [80,81]. However, to fully capitalize on the potential of these apps, there are important lessons in design, implementation, and evaluation. Public health officials who wish to design new or improve existing web-based apps for this purpose should ensure that outbreak detection is automatic and signals are verified by users [37,39,41-48,51,52,55,56], the app is easy to use [37,38,41-47,49,50,53,55,57], and staff and resources are available to support the operations of the app [27,37-39,44-52]. They should also conduct comprehensive and rigorous evaluations [37,40-47,54,56]. In addition, public health organizations should maximize the functionality of these web-based apps to support response actions and detection and notification. We recommend that future authors describing the development or implementation of mHealth web-based apps consider using the WHO criteria [82] to facilitate comparison across apps for outbreak responses. Further research is also needed on the development (with user needs assessments) and implementation (with segmentation for the personal, technical, and organizational factors affecting technology adoption, including the user environment) of web-based apps used in the control of infectious diseases. Finally, although evaluation studies were reported for 6 web-based apps [37-40,44-47,50-53,57] and some demonstrated a significant reduction in time from detection to notification [38-40], these were limited to process evaluations using data collected via the app. Our results suggest that the evaluation of web-based apps requires a more holistic approach for effectiveness evaluation. This includes using validated tools where possible and data from the user, the app, and the organizational environment (of the user and the organization hosting the app).

## Appendix

Multimedia Appendix 1 PRISMA (Preferred Reporting Items for Systematic Reviews and Meta-Analyses) checklist for this systematic review.

Multimedia Appendix 2 Summary of papers reporting on web-based apps for infectious disease outbreak response included in this review.

## Abbreviations

IT: information technology
mHealth: mobile health
WHO: World Health Organization
