# Supplementary material for: Web-Based Apps for Responding to Acute Infectious Disease Outbreaks in the Community: Systematic Review
Source: JMIR Public Health Surveill. 2021 Apr 21;7(4):e24330. doi: 10.2196/24330 (PMC8100883; doi:10.2196/24330)
Supplement: Multimedia Appendix 2 [file publichealth_v7i4e24330_app2.pdf]

App name[a]	Author(s) and year	Stated purpose of app	Location and setting	Mechanism(s) for detecting outbreaks	Features to support outbreak response	App development or implementation-reported lessons learned	Formal evaluation reported? Yes or No (if yes, outcomes reported)
**Computer-Assisted Outbreak Detection—SmiNET**	Cakici et al (2010) [41]; Kling et al (2012) [42]; Rolfhamre et al (2006) [43]	Improve real-time identification of outbreaks based on national data from SmiNET	Sweden; National to regional public health authorities	• Five different statistical algorithms:<br>○ SaTScan Poisson<br>○ SaTScan space-time permutation<br>○ Regression analysis (Farrington [59])<br>○ Outbreak<br>○ Simple threshold	• Automatic notification to public health staff of outbreak signals for further follow-up	• Used free open-source software for app development—low cost<br>• Verifies the signals seen in routine surveillance and potentially saves time<br>• Need to develop functionality for exporting data into analysis programs<br>• No user-based evaluation	• No
**Argus**	El-Khatib et al (2018) [38]	Improve the timeliness and completion of routine surveillance data	Central African Republic; National to regional public health authorities	• Not reported	• Automatic notification to public health staff of outbreak signals for further follow-up	• Simple and low cost<br>• Staff not accustomed to using smartphones for surveillance required several days training<br>• Predefined data entry forms easy to adapt to surveillance needs<br>• Technical and operational support offered by WHO[b]<br>• Two full time staff members needed for local implementation: one for IT[c] support and one to manage the server<br>• Purchasing and installing solar panels to generate power for laptops/phones helpful<br>• Booster training needed for users before launch	• Yes—pre/poststudy [38]<br>• Pilot data collected for 15 weeks in control versus pilot district and compared with previous years' data<br>• Median completeness of weekly reports significantly higher in pilot district in 2016 compared with 2015 (81% vs 29%; *P*<.01)<br>• Significant positive reduction in time to reporting over study period for pilot district between 2016 and 2015 (50% vs 19%; *P*<.001)<br>• Evaluation focused on technical usability not factors to assist in implementation and use

App name[a]	Author(s) and year	Stated purpose of app	Location and setting	Mechanism(s) for detecting outbreaks	Features to support outbreak response	App development or implementation-reported lessons learned	Formal evaluation reported? Yes or No (if yes, outcomes reported)
**SurvNet @Robert Koch Institute**	Faensen et al (2006) [44]; Hulth et al (2010) [37]; Krause et al (2007) [45]; Salmon et al (2016) [46]; Straetemans et al (2008) [47]	Comprehensive national system for communicable disease reporting	Germany; National public health authorities	• Algorithmic modeling<br>• Statistical modeling [61,62]	• Automatic notification to public health staff of outbreak signals for further follow-up	• Additional training and support tools needed<br>• Rigorous evaluation needed<br>• Difficult to implement system changes across the country<br>• Multidisciplinary approach to development of app helpful (eg, involve IT and medical staff)<br>• Keep data entry fields to minimum<br>• Clear language used within app<br>• Important to train users<br>• Need operating procedures to assess signals<br>• Progressive scale-up to the development of app helpful<br>• Increased awareness over time of need to monitor signals<br>• Reporting delays still evident<br>• Potential for missed outbreaks, if emergence is slow over time	• Yes—cross-sectional data [45]<br>• Median delay from notification to detection of outbreak reduced from 4 days to 1 day (2002-2005; regional level, not national level)
**Integrated Crisis Alert and Response System**	Groeneveld et al (2017) [56]	Improve the real-time identification of outbreaks and timeliness of response	Netherlands; Health district: hospital/GP[d] /out-of-hours GP	• Algorithmic modeling<br>• Statistical modeling (cumsum)	• Automatic notification to public health staff of outbreak signals for further follow-up	• Small size or coverage of GPs in system<br>• No specific patient identifiable data collected<br>• Automatic extraction based on diagnostic codes—quick and easy<br>• Need to still improve detection models and include more historic data<br>• No user-based evaluation	• No

App name[a]	Author(s) and year	Stated purpose of app	Location and setting	Mechanism(s) for detecting outbreaks	Features to support outbreak response	App development or implementation-reported lessons learned	Formal evaluation reported? Yes or No (if yes, outcomes reported)
**Vesuv**	Guzman-Herrador et al (2016) [48]	Outbreak rapid alert system to enhance reporting and improve information sharing	Norway; National to local public health authorities	• Algorithmic (not further stated)	• Automatic notification to public health staff of outbreak signals for further follow-up	• Potential for underreporting of outbreaks<br>• Users at local level less aware of the system<br>• Failure to update information in the app	• No
**Statens Serum Institut automated outbreak detection system**	Hulth et al (2010) [37]	Automated outbreak detection	Denmark; National public health authorities	• Algorithmic modeling<br>• Statistical modeling [61]	• Notification to public health staff of outbreak signals for further follow-up	• Potential for underreporting-laboratory reporting voluntary; coverage and completeness an issue<br>• Evaluation of the algorithm<br>• Improvement in usability tools to assess signals<br>• Improvement in integration with the national system<br>• Automation of the signal alerts<br>• Increasing the frequency of algorithmic detection, that is, daily	• No
**National Institute for Public Health and Environment (RIVM) automated surveillance**	Hulth et al (2010) [37]	Automated outbreak detection	The Netherlands; National public health authorities	• Algorithmic modeling<br>• Statistical modeling [59]	• Automatic notification to public health staff of outbreak signals for further follow-up	• Potential for underreporting-laboratory reporting voluntary; coverage and completeness an issue<br>• Evaluation of the algorithm<br>• Improvement in usability tools to assess signals<br>• Improvement in integration with the national system<br>• Automation of the signal alerts<br>• Increasing the frequency of algorithmic detection, that is, daily	• No

App name[a]	Author(s) and year	Stated purpose of app	Location and setting	Mechanism(s) for detecting outbreaks	Features to support outbreak response	App development or implementation-reported lessons learned	Formal evaluation reported? Yes or No (if yes, outcomes reported)
**Early Warning and Response System**	Karo et al (2018) [49]	Enhance disease surveillance and outbreak detection in emergency settings	Myanmar; Regional public health authorities and field bases and locations	• Not stated	• Automatic notification to public health staff of outbreak signals for further follow-up<br>• Weekly alerts contained information on risk assessment, characterization, and outcomes	• Lack of mobile reception meant offline data entry common, leading to issues of timeliness with data entry during outbreaks<br>• Laptops with Wi-Fi more helpful for field data collection	• No
**Early Warning and Response System**	Sheel et al (2019) [50]	Enhance disease surveillance and outbreak detection in emergency settings	Fiji; 12 sentinel health care facilities	• Not stated	• Automatic notification to public health staff of outbreak signals for further follow-up<br>• Weekly alerts contained information on risk assessment, characterization, and outcomes	• Open source/off the shelf<br>• Quick and easy to implement<br>• Training support provided by WHO	• Yes—evaluation against CDC[e] surveillance system criteria [50,63]<br>• 88% (286 of 325) alerts were verified<br>• Weekly bulletins helpful<br>• System easily adaptable for local context<br>• Smartphone app easy to use<br>• Delays in reporting still evident<br>• Issues with data entry and reporting re: consistency
**Decision Support System for Response to Infectious Disease Emergencies**	Li et al (2013) [27]	Provide a flexible and efficient tool that can be used for infectious disease emergencies in the field	China; National public health authorities and field based when needed	• Not clear, threshold based on historical data	• Automatic notification to public health staff of outbreak signals for further follow-up	• Different types and levels of users assists with system management<br>• Investment needed to maintain software and hardware<br>• Patient confidentiality issue with a web-based system with multiple users	• No

App name[a]	Author(s) and year	Stated purpose of app	Location and setting	Mechanism(s) for detecting outbreaks	Features to support outbreak response	App development or implementation-reported lessons learned	Formal evaluation reported? Yes or No (if yes, outcomes reported)
**China Infectious Diseases Automated Alert and Response System**	Li et al (2014) [39]; Yang et al (2011) [51]; Zhang et al (2014) [52]	Early detection and rapid response to outbreaks of infectious diseases	China; National public health authorities	• Algorithmic modeling<br>• Time-series modeling (C3 algorithm CDC) [64]<br>• Spatial and temporal modeling	• Automatic notification to public health staff of outbreak signals for further follow-up Confirmation of signals and report back to China Infectious Diseases Automated Alert and Response System by users	• Quality of data entry determines quality/timeliness of outbreak detection<br>• No new hardware needed, updates to software only<br>• Increased workload to manage false-positive signals<br>• Challenging to maintain user management policies and train staff with very high number of users	• Yes—cross-sectional data [39]<br>• System not inferior to routine reporting system in terms of size, duration, or time to report outbreaks<br>• Significant decreases in time to report outbreak of any size from 10 days to 9.1 days (P<.01; 95% CI 8.7-9.5)
**Public Health Emergency Response Information System**	Liang et al (2004) [58]	Prepare for and respond to public health emergencies	China; National to local public health authorities	• Not stated	• Proposed notification to public health staff of outbreak signals for further follow-up	• Not stated	• No
**WHONET—SaTScan**	Stelling et al (2010) [53]	Semiautomated disease outbreak detection	Argentina; National to local public health authorities	• Algorithmic modeling<br>• Statistical modeling [60]	• Notification to public health staff of outbreak signals for further follow-up	• WHONET free existing software<br>• SaTScan well known and used<br>• Needs work to tailor software/system design to the context<br>• Need operating procedures for responding to signals	• Yes—cross-sectional comparison (data entered via app vs paper based) [53]<br>• SaTScan detected an additional 14 *Shigella* outbreaks than paper system

App name[a]	Author(s) and year	Stated purpose of app	Location and setting	Mechanism(s) for detecting outbreaks	Features to support outbreak response	App development or implementation-reported lessons learned	Formal evaluation reported? Yes or No (if yes, outcomes reported)
**WHONET—SaTScan**	Vinas et al (2013) [57]	Early detection of outbreaks (via laboratory surveillance)	Argentina; National to local public health authorities	• Algorithmic modeling Statistical modeling [60]	• Signal verification at national level and sent to appropriate public health authority for action	• WHONET free existing software<br>• SaTScan well known and used<br>• Needs work to tailor software/system design to the context Need operating procedures for responding to signals	• Yes—cross-sectional comparison (data entered via app vs paper based) [57]<br>• 26 out of 28 events considered to represent disease outbreaks were detected before hospital staff were aware of any increase in the number of *Shigella* isolates
**French Institute for Public Health Surveillance app**	Vaux et al (2009) [54]	Increased timeliness of reporting of LRTI[f] outbreaks to reduce morbidity and mortality	France; Nursing homes	• Algorithmic (not further stated)	• Notification to public health staff of outbreak signals for further follow-up	• Need feedback from users on data inputs/outputs to further develop the app<br>• Further evaluation recommended	• No
**Infectious Disease Surveillance System**	Widdowson et al (2003) [55]	Automated outbreak detection	The Netherlands; National public health authorities and participating laboratories	• Statistical modeling (regression adjusted for seasonality) [59]	• Notification to public health staff of outbreak signals for further follow-up	• Simple model for detection of events above historical threshold—can be adapted over time<br>• Need to improve coverage of participating laboratories	• No

App name[a]	Author(s) and year	Stated purpose of app	Location and setting	Mechanism(s) for detecting outbreaks	Features to support outbreak response	App development or implementation-reported lessons learned	Formal evaluation reported? Yes or No (if yes, outcomes reported)
**Adjustable Epidemiologic Information System**	Wu et al (2011) [40]	Provide a more rapid response to infectious disease outbreaks	Taiwan; Local public health authorities	• Algorithmic (not further stated)	• Automatic notification to public health staff of outbreak signals for further follow-up<br>• Public health action taken and reported to national public health authority	• Feedback from users needed<br>• Further rigorous evaluation needed	• Yes—cross-sectional time-series analysis with 3 years of Adjustable Epidemiologic Information System data [40]<br>• Significantly decreased mean personnel response time to an outbreak from 6.92 days (SD 22.11) in 2006 to 2.13 days (SD 5.77) in 2007 and 0.88 days (SD 1.52) in 2008<br>• Significantly reduced personnel system interface time over study period<br>• Reduced duration of clusters over study period—shortening trend (*P*=.019) that concurred with the shortening of personal response time from a mean of 64.8 hours per cluster (SD 47.3) to 25.2 hours per cluster (SD 38.2) (*P*<.001)

[a]The same app may appear on multiple rows; studies of the same app but for a different stated purpose or occurring in a different location or setting are reported in separate rows.

[b]WHO: World Health Organization.

[c]IT: information technology.

[d]GP: general practice.

[e]CDC: Center for Disease Control and Prevention.

[f]LRTI: lower respiratory tract infection
